# Extracts

**Published:** 1883-07

**Authors:** 


### Extracts.

---

Report of a Case of Battey's Operation.—(*N. A. Drake, M.D., in St. Louis Medical and Surgical Journal.*)—In December, 1881, I was consulted by an intelligent colored girl, 21 years of age, for painful menstruation.

She said she always suffered terribly during her monthly periods, so much so that she was obliged to be in bed half or two-thirds of her time. I gave her a superficial examination, and as she was not suffering then, asked her to see me again when menstruating.

Several days after she sent for me. I found her on the floor writhing in pain, which appeared to be spasmodic, simulating labor pains. She had not yet commenced to flow. I proposed to give her morphia hypodermically, but she said she could not take morphia, it made her so sick. However, I gave her one-quarter grain, guarded by one one-hundred-and-twentieth grain atropia, as per Bartholow. When I inserted the needle she went off into a convulsion. From appearance would call it hystero-epileptic. This was followed by a state of quietness that lasted for several minutes. She came out of it with one of those paroxysms of pain.

I was informed she would have such convulsions many times a day during menstruation; and when she was well she would have them on the least excitement, mattered not where she was, she would fall down in a fit.

I used all the external applications usual in these cases, to cause her to menstruate, viz: hot hip baths, foot baths, heat locally, hot vaginal injections, etc., etc.

The nausea did follow the use of morphia. It seemed to me I never saw more obstinate nausea; the stomach would retain nothing for some time; but after many repeated doses of cerium she got better. This state of things continued until the menstrual flux became established. The stomach, however, remained very irritable, and the pain continued, although not so severe.

I think I gave her more relief by small doses of atrophine hypodermically than from anything else.

When she got around from this she came to my office for an examination.

I found her, for her class, a very intelligent girl. Had lived for several years in one of our best families in the city.

Her history, as related by herself, is about as follows: She commenced menstruating when about sixteen years of age, and it was always painful. More at times than others.

Had had these fits since she commenced to menstruate; has falling of the womb, and had worn a pessary for nearly a year. The stomach had always troubled her. Had had all the diseases imaginable, to let her tell it. Had been catheterized for nearly three years, nearly all the time. Sometimes just before menstruating could pass water for a few days. Was paralyzed for several months a couple of years previous. Could not walk for some time.

On examination I found a closed bar pessary in the vagina, and it was nicely adjusted. Removed it, and after a thorough investigation could find nothing wrong only the uterine displacement and a prolapsed ovary. Some cystitis, as a matter of course, but from analysis of urine could find no disease of kidneys.

After considering some time, I put her upon a constitutional treatment, which amounted to but little, as her stomach would not tolerate anything any length of time. Gave bromides, and they were retained till her fits were not quite as often. The lady with whom she lived catheterized her every day, or every other day. She had learned, if she went without drinking, she could go three or four days without having catheter passed.

You will at once discover that the hysterical element in the girl's nature largely predominated, although I saw her in some of her convulsions when they had more the appearance of epilepsy.

Her next menstrual period was worse, if possible, than the previous. I used atropia in larger doses, and finally by combining a little morphia with it, succeeded in controlling pain to a great extent.

It is not necessary for me to follow the case from month to month, as each menstrual period was, practically, a repetition of the other.

I became convinced that medical treatment would do her but little good, and after considering the case from every standpoint, I advised the removal of the ovaries, or marriage, expecting pregnancy might be beneficial.

The latter course she declined, and after a short time to consider it, she accepted the former, after I had stated to her plainly the dangers of the operation, and the chances of a cure if she should get well of the operation. She was anxious to take the chances, as she could expect no permanent effect from medical treatment after having apparently tried and exhausted nearly all known therapeutic measures.

Prof. Hegor, of Germany, probably was the first surgeon to remove the ovaries for diseased conditions, July 27,1872.

We read of the amorous Lydian Kings having their concubines spayed, so they might preserve their beauty, etc., etc., and to satisfy their insatiable lust; and we also read of a Hungarian *sow gelder* spaying his own amorous daughter.

But, as stated above, Prof. Hegor was probably the first man to do the operation legitimately, and it was a few days later, August 2, 1872, done by Lawson Tate, of England. Prof. Hegor first did the operation to arrest the growth of fibroid tumors of the uterus. His reasons for it were evident. It had been observed in woman with fibroid tumors that after the change of life the tumors ceased to grow, and in many became smaller. He conceived in this that the life of the fibroid tumor was the blood supplied by the monthly congestion. Hence, if he would remove the ovaries he would produce premature change of life and thus stop the growth of the tumor.

Upon these hypothesis he acted, and he reports quite a number of cases in proof of the correctness of his theory. And also the operation being far less dangerous than the eneucleation of the tumor or the extirpation of the womb.

Other operators have followed him, and all corroborate the correctness of his views.

But to Dr. Battey, of our own country, are we indebted for the operation for the cure of dysmenorrhœa, epilepsy and mental diseases bordering on insanity, after all other means and remedies have failed.

(Dr. Battey operated in August, 1872, a few days later than Dr. Tate; but he, Battey, published his cases to the world, while Drs. Hegor and Tate did not publish theirs. Hence Dr. B.'s claim to priority.

Probably in no country has gynæcology made so rapid strides in the past twenty-five years as in America.

The profession at home concede to Dr. Battey, of Rome, Ga., a front rank for this operation which rightly goes by his name.

In Europe it is not an acknowledged operation for dysmenorrhœa, and we have some surgeons among us who are yet unwilling to accept any great operation unless it is brought across the water. I have heard Emmet's operation condemned, and yet for all of that it has taken its place as an acknowledged operation, even among English surgeons, who are not willing to accept originality from the United States. And Battey's operation is forcing its way to an acknowledged place in the same way.

Dr. Goodell says, and his authority is unquestioned: "The operation of spaying is yet but in its infancy, and time is needed to develop its resources. But I cannot help feeling that in carefully selected cases it will prove the sole means for curing many mental and physical disorders of menstrual life, which have hitherto baffled our science, and are a standing opprobium to our profession."

What I hoped and expected to gain by the operation: Cessation of menstruation directly. Although tabulated cases by Emmet and Goodell give a small per cent. of menstruation, so called, after operation; but I cannot look upon it other than a flux, and thus to relieve the terrible monthly pains, and through this to cure the hystero-epeleptic convulsions.

The mortality from the operation is not greater than from other capital operations. Agnew, in his work, gives a general summary of 171 cases, 139 recoveries and 32 deaths, or 18.72 per cent. Of the 171, 144 were by laparotomy, of which 117 recovered and 27 died, or 18.75 per cent. 27 were by elytrotomy, with 22 recoveries and 5 deaths, or 18.57 per cent.

You see by this, the operation through the vagina gives the smaller death rates. But remembering the experience of Goodell, where he was obliged to do abdominal section after having made the incision in the vagina and found the ovaries beyond his reach, being bound down by adhesions, I concluded it would be advisable to go through the abdominal walls. In this I had the concurrence of Drs. Cathcart and Mitchell, after taking into consideration all the chances of there being an adhesive inflammation.

After quite a number of delays, owing to her irregularity in menstruating and the irritable stomach, on April 29, 1882, assisted by Drs. Cathcart and Mitchell, and Mr. Burgett, medical student, I removed her ovaries by abdominal section.

I will not take your time in describing in detail this operation; suffice it to say, the ovaries were easily reached through a three inch incision; ligated them with carbolized silk ligature; closed the wound with silk worm gut ligature, and dressed with carbolic cerate and absorbent cotton, with flannel binding around the body. Used carbolic acid as an antiseptic. All sponges, instruments, ligatures and water used were all carbolized. Used ether as anæsthetic. She rallied well from the operation. Got union by first intention, and she got along for three weeks without an unfavorable symptom.

About this time she began to sit up, and circumstances over which we had no control compelled us to move her to another house, the sanitary surroundings of which were not the best. She sat up part of the time, and everything appeared to be swimming along nicely, when she took a violent chill. I expected to find some peritoneal trouble, or some inflammation caused by the operation, but could detect nothing of the kind.

Gave quinine in anti-periodic doses, but our old enemy, the stomach, refused to tolerate it. The chills returned every 24 hours, and the fever was constant. I soon became convinced I had a veritable case of malarial fever. I resorted to quinine hypodermically, but it was difficult to get enough into the system in this way to do any good without causing abscesses.

Finally I resorted to suppositories of quinine. This did well for a week or ten days, when the rectum rebelled. But I rung the changes as best I could on these three modes, when, after a struggle of three weeks, she gradually began to improve.

On trying to get out of bed she claimed she had no use of her right leg, and surely there was a certain degree of paralysis. I insisted on her getting around, had a pair of crutches made for her, and fairly forced her to use them. She improved rapidly, and I had ceased visiting her but occasionally, when I was summoned again, and found her suffering from dysentery. This lasted for a couple of weeks, when it gradually subsided, but left her with external hemorrhoids. These I laid open and pressed out the clot of blood, and I heard but little of them after that. From that time until the present, January, 1883, her improvement has been uninterrupted.

She is now capable of taking care of herself. Somewhat lame yet, but I see no reason why she should not entirely recover. The bladder trouble still remains, but not so obstinate.

I am treating it with faradism with an insulated electrode in the urethra and bladder, and the positive pole in the inguinal region. She will pass her water for two or three days after each seance.

*Results.*—She has had, several times, a slight bloody discharge, no pain with it. Hence the dysmenorrhœa has never returned. Occasionally, when she gets very nervous, she will have a convulsion. But she does not have one now where she used to have fifty. All in all I consider the results highly gratifying, although we encountered obstacles we never dreamed of. But I believe the amount of good ascomplished is worth to her all she has undergone, and she so expresses herself.

I am convinced she will continue to improve, and hope soon to get her in condition to urinate all the time, herself. But so many surprises have occurred in the case that I shall not be surprised at anything that may yet occur. The case has been an interesting one all the way through, to me, and is one that puts a man to his wit's end, often, to meet the emergency.

Thinking that the many interesting points might elicit a discussion that would be interesting and beneficial is my excuse for reporting it.

At this date, April 11, 1883, the woman is comparatively well. She urinates nearly all the time herself. I have about ceased to use electricity. She comes to my office occasionally yet, for it, more to relieve the pain in her lame leg than anything else.

Sometimes she will have a slight uterine discharge of blood. Once quite freely while suffering from a severe cold.

Since preparing the above paper, I have read what Lawson Tait has published in the *British Medical Journal.* In that he contends that we are all mistaken about the organs that control menstruation.

We have all been taught that the ovaries presided over this function, but he claims the fallopian tubes are the organs that control menstruation, and proves it to his satisfaction by removing the tubes with the ovaries, and has no flux at all after it.

This would seem to be evidence conclusive, but we must remember that the greater number where the ovaries only are removed, have no discharge after it. We all admire the success and boldness of Tait, but I must confess that I am not yet far enough progressed—excuse the term—to accept his theory of menstruation.

Somehow our native gynæcologists do not have the wonderful success of Tait. In his last thirty-five operations for the removal of ovaries and tubes, he has not had a single death.

We feel proud of our eminent Battey, Thomas, Bozeman, and a score of other American operators, but none have arrived to his point of excellence.

Circumstances, of course, must be different. We live in progressive days, and we have no fears that our Yankee nation will be left very far behind.

---

The Frequent Repetition of Doses.—Under this heading we find in *New Remedies* an extract from a lecture of Prof. A. A. Smith, in the Bellevue Hospital Medical College of New York. The following are some of the Doctor's suggestions:

*Chlorate of Potassium.*—Grain doses, given every half hour, in scarlet fever, diphtheria, tonsillitis, etc., will produce the same results as larger doses, without the danger of the evil effects resulting from the accumulation of the drug in the system, as sometimes happens when it is administered in the ordinary way. Indeed he believes they will produce better results upon the inflammations of the throat.

*Croton Chloral*, when used to relieve neuralgia, is more efficacious when a grain is administered every half hour until the symptoms abate, than when, as is usually the case, five to eight grains are repeated every two hours until fifteen grains are taken. Another advantage of the former plan is that it avoids the irritation of the stomach so apt to be caused when the larger doses are used.

*Jaborandi*, given in five to ten minim doses of the fluid extract, will produce diaphoresis without depressing the heart's action—an effect which has so often followed the use of larger doses as to cause many physicians to refrain from the use, especially in the puerperal state.

*Sulphate of Atropine.*—One-hundreth of a grain, dissolved in a glassful of water and given in teaspoonful doses every half hour or hour, is a valuable remedy in spasmodic croup. An emetic or purgative should precede it when the condition of the stomach or bowels demands.

*Bromide of Sodium.*—A few grains dissolved in a tumblerful of water, so that each teaspoonful may represent a half grain, will quickly quiet the nervous disturbance of teething infants, or fever not dependent upon the onset of an inflammation or other grave trouble, but rather such as may follow excitement of any kind. The dose should be repeated every ten or fifteen minutes.

*Chamomilla.*—A minim of the tincture given every fifteen or twenty minutes is a better sedative than chloral hydrate for sleepless children or those who are easily disturbed by noise or other causes. A simple way of administering is to put a teaspoonful of the tincture in a glass half full of water, and let the child drink it at its pleasure.

*Ipecac.*—A drop of the wine of ipecac, repeated every ten or fifteen minutes, will often relieve obstinate vomiting from different causes, and will sometimes also check diarrhœa. Given in this way in water it does not nauseate.

*Nux Vomica*—A drop of the tincture given every ten minutes will often produce most marked relief in sick headache not of neurotic origin. It should be given immediately or soon after meals.

*Digitalis.*—A single drop of the tincture given to a patient suffering from symptoms due to heart disease, where digitalis is indicated, administered at intervals of an hour, or half hour, according to the severity of the symptoms, will often give greater relief than larger doses, and without liability to ill effects.

*Castor Oil.*—For the diarrhæa of children, accompanied with slight inflammation, straining, and the passage of jelly-looking matter, but not true dysentery, five drops of castor oil given every hour with sugar and gum is an excellent remedy.

*Pulsatilla.*—Two minim doses of the tincture, repeated every hour, are said by an authority in venereal diseases to have given greater and more rapid relief in cases of orchitis and epididymitis than any other treatment. Professor Smith also vouches for its efficacy, when administered in this manner, in dysmenorrhœa not of a membranous, obstructive, or neuralgic character.

*Calabar Bean.*—One-fiftieth of a grain of the extract, repeated every half hour for six or eight doses, is an effectual remedy for the flatulence and a sensation of palpitation or fluttering at the pit of the stomach, suffered by many women at the menopause. After the number of doses above mentioned have been taken, three hours should intervene before recommencing its use.

*Aconite*—One third to one-half minim, given every fifteen minutes, will lessen fever (when it is not the commencement of one of the continued fevers) when the skin is hot and dry, the pulse full and bounding, and the mucous membrane of the throat and nose dry. When so used it will also arrest a "cold in the head," and is useful in cardiac hypertrophy with palpitation, severe headache, and disturbances of the nervous system due to increased force of the heart-beat.

*Hamamelis.*—Two minims given every half hour will often control hemorrhage from the nose, uterus, and from hemorrhoids.

*Belladonna.*—Minim doses given every half hour is a good remedy in nasal catarrh and bronchitis, accompanied with free secretion. Owing to its tendency to cumulative effects, it should be suspended for a time after eight or ten doses are given. Thus administered, it retards the exudation of serum and sustains the heart's action in pulmonary œdema.

*Chloride of Ammonium.*—Two grains, combined with ten or fifteen minims of the tincture of cubebs, given every half hour, oftentimes controls acute pharyngitis and superficial inflammation of other tissues about the throat. When the pharingitis depends upon a gouty diathesis, add to the above mixture ten minims of the ammoniated tincture of guaiacum and administer every hour.

*Citrate of Caffeine.*—One grain, every half hour, often produces most marked relief in migrains.

*Gilsemium.*—Three-minim doses of tincture, every half hour, will often relieve miraculously neuralgias about face or head, and have no ill effects.

*Guarana*—Fifteen minim doses of the fluid extract, given every fifteen minutes, frequently relieve headaches, especially when they are periodical, and not of malarial origin.—*Medical Truth.*

---

One of the Causes of Hæmoptysis.—*J. Y Totherick, M.D., M.R.C.P.E., in Birmingham Medical Review*—Some three years ago a young gentleman who, three months previously, had been attacked by hæmoptysis, became an inmate of my house. He was a youth of bright intelligence, and vigorous intellect, and happy, cheerful temper. He had a good family history, and came of a long lived race. No trace of struma or phthisis was to be found in its records. From the age of 14, when he was a boy rather small for his age, he rapidly grew until he was nearly 5 feet high. Then came the hæmoptysis, which both he and his friends attributed to over exertion on a bicycle. When he came to me he was considered quite well, but rather weak from having grown too fast. The most rigid and careful examination by percussion and ascultation could detect no morbid or exaggerated sound within the chest walls. The attack of hæmoptysis appeared to have left no ill effects behind. The thorax itself was perfect symmetry. Repeated examination revealed no lesion. There was no cough, no increased temperature, no dyspepsia, no emaciation. The time was summer, the weather genial, the climate all that could be desired. Without any warning, and apparently without any cause, shortly after a little drive, hæmoptysis to a considerable extent recurred. No fever, however, supervened. For a few days there was some expectoration of little dark clots and he was well again. A month passed and our fears were subsiding, when another but slighter attack came on, and the recovery was neither so rapid nor so perfect. A little dullness to percussion was found at the angle of the right scapula. The temperature for a short time was increased at night, and the appetite somewhat failed. Nevertheless, in a few weeks he was well again, there was no cough or expectoration, there were no morbid sounds, and although it was apparent that he had lost some ground, perhaps as much through apprehension as anything else, he was once more in very fair health. Three months more passed, and the weather was colder and damper, he had ceased his daily rides and drives, he had increased in weight and healthy look, when, without any warning, bleeding again came on, slight at any one time, but recurring every few days. Now dull patches of lung became perceptible to the ear, moist sounds rattled in the bronchi, cough became persistent, fever supervened, appetite failed, pleuritic pains occurred in various parts of the chest, emaciation, perspiration, perulent expectorations, diarrhœa, signs of cavity, all followed in due order, and death at length made an end of the sad story.

I have related this case, not because there is anything very striking or unusual, but because I consider it typical of a large class of phthisical cases, and because it came under my own immediate care and constant supervision, and gave rise in my mind to a series of thoughts as to the originating cause of the hæmoptysis, and perhaps the resulting phthisis in the type of cases I refer to.

The cause of hæmoptysis in similar cases has, I think, become more and more clear to my own mind during a succeeding period of much more extended observation; and, although I have never as yet been able actually to demonstrate the truth of the theory I hold, I still venture to place it before you for your consideration and discussion, and whatever interest attaches to an original theory in medicine, attaches itself so far as I am aware to this.

The type of cases is, I think, sufficiently indicated by the one I have related, viz: phthisis occurring in youths, male or female, who have very rapidly shot up in growth in the past two or three years. I suppose few of us doubt that hæmoptysis, *i e*, the actual expectoration of blood due to pulmonary lesion is at any rate a frequent mode of the onset of phthisis, and that the bloood extravasated during the attack, but not got rid of by expectoration may be the efficient cause of local inflammation of lung tissue —ending eventually in cavities. But I believe we must also take into consideration the fact that blood may be extravasated into lung tissue without getting into the bronchi, and consequently without hæmoptysis, and nevertheless lead to equally fatal results. It is not the blood which is expectorated—but that which remains behind—acting as a foreign body in the centre of inflammation which does the mischief. In either case, however, whether the blood is expectorated or remains in the areolar tissue, capillaries must be ruptured, and it is one of the causes of this capillary rupture that I want to suggest to your notice.

Now when a youth shoots up, it is as a matter of course his bones which are lengthening, most strikingly the bones of the legs and the arms—the long straight bones. In fact they often appear to grow out of all proportion to, and even at the expense of the other tissues, such as muscle and fat. But as a matter of fact, whilst the long straight bones are most rapidly growing in length, the long curved bones are growing in equal proportion. And the result is that the ribs are forming a much more capacious receptacle for the organs they contain. The girth of the chest increases in proportion to the length of the ribs. Now taking this fact, together with the other fact that it is during or very soon after the rapid growth that phthisis begins so frequently in the class of cases I am speaking of, accompanied either by hæmoptysis or perhaps by invisible extravasation of blood into the lung tissues from rupture of the capillaries, I think there is good reason to suppose that rapid growth in the capacity of the chest walls and ruptured capillaries are closely connected together. For observe what is the accompaniment—I do not say the consequence—of this rapid growth. All the tissues we can see are highly attenuated. The muscles become longer and thinner, the areolar tissues become less thick, the skin is stretched to cover the extended surface. As I have said before, the bones appear to grow at the expense of all the other tissues. If this is so externally there is good reason to suppose that it is so internally, also, and therefore, that in the lungs, the blood vessels and the bronchi all become for the time attenuated and weakened by the rapid growth of the bones, and if so weakened, more liable to be ruptured. But within the chest walls there is still another reason for suspecting that this attenuating action of rapid growth may be more powerfully operative than elsewhere. The old saying that nature abhors a vacuum will serve to explain my meaning. The thorax being practically a shut cavity, when its walls rapidly expand by the growth of the ribs, its contents must expand to fill up that cavity or the abhorred vacuum would result. Of course they may expand in more ways than one. No doubt in robust and well nourished constitutions the internal structures may grow *pari passu* with the external, and not being attenuated may not be weakened, the cavity being filled by normal growth. Again, the areolar tissue, which is very elastic, may stretch and become more abundant. But I think there is good reason to assume that in a certain proportion of cases of this rapid expansion of the chest walls by bone growth, the tissues both of the blood vessels and air tubes become stretched and attenuated in order to fill up the space which they are forced by this expansion to occupy; at any rate we often see these conditions co-existent, very rapid growth of young people of both sexes both in length of limb and girth of chest walls, together with marked attenuation of the muscles and other external structures. Also the frequent development of phthisis preceded or accompanied by hæmoptysis and local congestions, which you cannot account for in any way so satisfactorily as by supposing extravasation of blood from ruptured capillaries. And when we have blood in any part of the body external to the blood vessels it is essentially a foreign body, and liable as other foreign bodies to give rise to inflammation and all sorts of fatal mischief. I do not mean to deny the influence of tubercle in giving rise to hæmoptysis, only I think this influence is often overrated and other causes ignored.

Well, then, supposing that there is any truth in my theory, that in this rapid growth we have these delicate vessels rendered still more delicate by too rapid and imperfect nutrition, and attenuatdd by being stretched out to occupy too quickly the greatly enhanced capacity of the thorax, it seems to follow that they may be very easily ruptured by causes which would produce no harm in a normally healthy and slower growing lung. An ordinary catarrh with its troublesome cough, an extraordinary spurt of physical exertion, loud shouting or continued singing, or a hundred causes which one could easily name, might produce the hæmoptysis, or extravasation, or both, which so often ends in a fatal phthisis. Such I believe to be the history of the commencement of many cases which we are constantly meeting with.

I do not for a moment pretend that this theory will account for all cases of phthisis or even of hæmoptysis from lesion of lungs; no theory will—certainly not the bacillus theory of Koch. I only suggest it as a theory sufficient to account for a certain class of cases. I have said that I cannot at present demonstrate its truth; to do so would necessitate a rather difficult combination of circumstances. One would have to find a case such as I have related, fatal in its early stage from some totally different disease or accident, and make careful preparation of the lung tissues to compare with other lung tissue obtained under other conditions. Although on the lookout for many years, I have not been able to accomplish this. I only advance this theory as one step further back in the history of hæmoptysis, and of hæmoptysis as a cause of phthisis. The theory easily squares with heredity, as I suppose it is as common to find tall sons of tall parents as to find consumptive fathers of phthisical sons. And if the theory should prove a true one so far as it goes, I think it opens out a prospect of that prevention which we all agree is so much better than cure, in the careful management and nutrition of the rapidly growing young. At any rate I leave the discussion of it in your hands, with a simple hope that some little step may be gained towards truth.

---

Some New Discoveries in Regard to Erysipelas.—(*Detroit Medical Age.*)—In a paper read before the Cincinnati Medical Society (Lancet and Clinic), Dr. Joseph Eichberg gives a resume of a treatise on the etiology of erysipelas by Fehleisen, of Berlin, which treatise he regards as another step in the gradual perfection of our knowledge of the disease. He refers to the various theories of the causation which have obtained, beginning with Galen, who referred the cause to disturbances of the biliary secretion, and continuing down the line to Huter, who advanced the theory that the erysipelatous virus belonged to the class of micro-organisms. Subsequent investigations have confirmed the theory to demonstrating the presence of micrococci and bacteria. The author differs from Huter, who considers the virus to be small micrococci in active movement, while he lays special emphasis on the fact of their immobility.

Fehleisen's experiments succeeded in isolating the erysipelas micrococci and in propagating them by culture, producing in this manner in the course of two months, fourteen generations. In the manner of their growth they presented peculiarities which at once enabled him to distinguish them from the micrococci of pyæmia and other affections whose germs are morphologically identical with those of erysipelas. The inoculation of rabbits with these artificial culture fluids produced a disease absolutely identical with erysipelas. Patients in the hospital were also inoculated with identical results. In selecting patients for these experiments a double purpose was sought to be accomplished. Remembering the frequent mention in the literature of the subject, of the favorable influence exerted by a concurrent attack of erysipelas in cases of neuralgia, typhoid fever, acute rheumatism, chronic diseases of joints and various forms of syphilis, lupus and many neoplasms, five of the patients selected for the experiments were affected with morbid growths and two with lupus. In six of the seven cases erysipelas was promptly developed; the seventh case had had numerous previous attacks of erysipelas, the last occuring but three or four months previously, and was supposed to have thus established a tolerance for the virus. Without considering each of these cases in detail, it may be stated that the development of erysipelas in no case did harm, while in three the therapeutic effect was quite satisfactory. Such inoculations are, however, permissible only when hope of benefit from operative interference has passed.

Aside from their thereapeutic effect, these experiments are worthy of consideration in deciding the question of the origin of erysipelas. All cases were types of pure erysipelas as determined by Bergmann, who examined them all in common with many of his colleagues of the Wurzburg clinic. In regard to the researches of Lukomsky, Billroth, Ehrlich and Tillmans, who found the micrococci in the lymphatics of the skin and subcutaneous fat, and in the blood vessels, liver, kidney and substance of the heart, it may be safely presumed that in these cases there was a complication with pyæ or lymphangitis or phlegmon. In the uncomplicated affection the micrococcus is found only in the lymphatics, which is characteristic of the affection. The spread of the disease does not occur, as in lymphangitis, along the course of the lymph stream, but the dissemination takes place in all directions, frequently against the direction of the lymph current.

With reference to the spread of the disease in any community there can be no doubt that it is contagious, *i. e.*, transmissible from man to man by direct contact, through the use of instruments, etc., but this is the only or even the usual method of dissemination. On the contrary, no reasonable doubt can be entertained that the micrococci multiply and generate outside of the human or animal body. Morever it is not an easy matter to produce an artificial erysipelas without resorting to the method of cultivation outside of the human body. Many experiments of direct inoculation from man to man have given negative results, which prove that the danger of contagion from a person suffering with erysipelas is not very great. The bacteria which have entered the body, disappear almost as quickly as they multiply, without ever reaching the surface, and thus having opportunity to act, as the means of secondary infection. The micrococci of erysipelas would then very speedily disappear altogether were there not some soil in which they might develop, other than the human body. As pointing to such a conclusion, there may be cited the fact that in artificial cultures they multiply when cultivated upon potatoes, as well as upon coagulated blood serum or gelatin.

Another interesting feature of the experiments bears upon the question of immunity from second attacks. After a primary inoculation, seven persons were vaccinated; six were affected with erysipelas, the seventh patient had frequently been affected and had passed through his last attack a few months prior to the experiment. Of the six other successful vaccinations, two were repeated several times. The third case, successfully, on the 7th of October, subsequently on the 1st and 9th of November, unsuccessfully. In case No. 3 patient had erysipelas in December, 1881; on the 7th of October, 1882, she was successfully vaccinated with the culture virus; on the 9th of November, thirty-three days after this, the vaccination was unsuccessful. We may conclude from this that one attack of erysipelas confers an immunity of short duration from later attacks.

The author concludes his paper by reporting some experiments made with a view of testing the effect of two autiseptic agents upon the disease germs. The two agents were those used for the dressing of wounds in Bergmann's clinic, a one per cent solution of corrosive sublimate and a three per cent solution of carbolic acid. After exposing the germs on a platinum wire to the action of the carbolic acid for twenty seconds, no apparent effect was produced; for the artificial cultures developed as rapidly and extensively as before. An exposure of thirty seconds caused an imperfect and retarded development of the cultures; an an exposure of forty-five seconds destroyed them altogether. The solution of corrosive sublimate destroyed them much more quickly, an exposure of ten to fifteen seconds being sufficient to prevent their development on gelatin. As showing the value of antiseptic dressings, suggested by these experiments, the author cites the statistics of the surgical clinic of Bergmann, where, during a period of four and a half years, erysipelas occurred only in two cases treated with the antiseptic dressing, and he adds, this very limited number may be ascribed to some slight defect in the dressings; and when it is remembered that erysipelas is of very frequent occurrence in Wurzbug, these figures show decidedly in favor of the antiseptic method. When it is further remembered that many cases of operations about the face and head, where the antiseptic dressing was not applicable, were during the same time attacked with erysipelas, any additional proof seems unnecessary. The antiseptic dressing will, however, only prove efficient when its application has been preceded by careful disinfection of the wound and of surrounding parts; for this purpose strong solutions of carbolic acid answer best, as they penetrate somewhat into the tissues around the wound, without, at the same time, coagulating the albumen of these tissues; an objection which militates against the employment of corrosive sublimate.

As far as erysipelas is concerned, the labors of Fehleisen seem to decide conclusively a great deal that has hitherto been only speculation and surmise; and, with reference to completeness, are really more satisfactory than the valuable discovery of Koch which they so briefly follow. How far the future physician is to be benefitted by this work in the field of therapeutics it were impossible to conjecture. It is the first time that artificial culture fluids have been successfully used for the production of disease in man, and the very success which has crowned these efforts will probably serve as an encouragement to many to follow in the path which the author has so brilliantly indicated. We can only hope for the sake of humanity and of our science, that those who may come after shall, like Fehleison, bring to their work scientific acumen, clear observation, and above all and over all, a sincere desire to relieve suffering and ameliorate distress.

---

American Medical Association.—The thirty-fourth annual meeting of this association was called to order at Cass Hall, in Cleveland, Ohio, at 10:30 on the morning of the 5th, by Dr. X. C. Scott, chairman of the committee on arrangements. He introduced the Rev. Richard Gilmour, Bishop of Cleveland, who introduced a very appropriate innovation in this part of the programme, by giving, instead of the lengthy prayer which has heretofore been thought necessary, a few introductory remarks and closing by repeating the Lord's prayer. Dr. Scott then formally introduced the president, Dr. John L. Atlee, of Pennsylvania, "a man renowned with spotless reputation, and one of the noblest ornaments of the profession." Dr. Atlee, after bowing his acknowledgment of the repeated rounds of applause with which his venerable form was greeted, introduced General Ed. S. Myer, who delivered the address of welcome. The eloquent soldier, after paying a glowing tribute to the medical profession, welcomed its representatives, as they had come up to their annual gathering, to the hospitalities of the city. Dr. Scott then announced the programmes of the day for the different sections, and after mentioning the points of interest in the city, announced still further that no one who could not sign the code of ethics of the association would be admitted as a delegate.

Dr. Atlee then read the Presidential Address. After thanking the association for the honor it had conferred on him, he expressed deep regret at the absence of the New York delegation. He hoped, however, that this absence would be but temporary, and that at the next meeting every State of the Union would be represented. Specialties are now the order of the day, and although he was far from favoring them on ordinary occasions, he felt constrained on the present occasion to defer the fashion. He, therefore, would present his specialty. It was a rare one; a geaduate of 63 years' standing. Such a specialty, he felt, absolved him from any effort at a scientific paper, and made it his duty only to give some reminiscences of his professional life, begun so many years ago. He then gave a recital of the events of his college life, commencing with his first course of lectures in medical department of the University of Pennsylvania, in 1817-18 His reference to his teachers was both pleasing and touching. They embrace names which still live and are incorporated in the history of American medicine: Caspar Wistar, Jno. Syng Dorsey, William E. Horner, Hugh L. Hodge, John Redmond Coxe, Thomas C. James (professor of midwifery, of Quaker origin, and of such delicacy as prevented him from lecturing on the female generative organs), Nathaniel Chapman and Philip Syng Physick, the great American surgeon. "A pupil of John Hunter, Physick taught the doctrines of that great man. As I recall his course of lectures, it seems to me that he was one of the most impressive teachers that I have ever listened to. Dr. Physick was remarkable for his great attention to details, and in his operation upon the cadaver he carefully observed all the rules for operating upon the human body. He also recapitulated the lecture of the preceding day before going on with his subject, by questioning the students who occupied the first two rows of seats in the ampitheater. I may refer to one incident which may illustrate his method and his carefulness. On one occasion he stumped the whole class; he had been lecturing on lithotomy the preceding day, and he put the question to the first student. 'What instrument should be provided for the occasion?' The answer appeared to have been correctly given But he was not satisfied. The question was repeated to the next student, and finally to the whole class, with the same result. Dr. Physick then said it was 'a pin, gentlemen, a pin,' that was needed to complete the list. This showed his precision, and impressed upon us the necessity of taking care never to go to an operation without the minutest preparation.

Dr. Physick was a man of medium height, with very regular features. His face at that time was pale, as if he suffered from delicate health. He was of very abstemious habits. I remember on one occasion, at a party given at his house, when the servant brought in a tray with wine, I was standing beside Dr. Chapman, when I placed my hand upon a decanter, as I supposed, of wine; Dr. Chapman touched my elbow, and told me not to take that; I filled the glass from another bottle, and afterwards asked the doctor why he had checked me; he said the first was simply colored water that Dr. Physick had provided for his own use.

One of his favorite precepts was to insist upon great attention to diet after surgical operations. I may mention this anecdote. In one of his lectures he spoke of a very important surgical operation, and said that there was a necessity for attention to absolute diet. The next day in recapitulating, he asked a student what was meant by absolute diet. The student said, 'toast or water.' 'Will any gentleman tell me what is meant by absolute diet?' appealing to the whole class. There was no reply. 'Water, gentleman, water.' A precept I have never forgotten, and which, I think, is not sufficiently observed at the present day after important surgical operations."

In referring to the practice of these days Dr. Atlee said:

"It was the time of calomel and the lancet. With regard to the one, I need not speak; but of the latter I feel well assured that the almost total disuse into which it has fallen has cost many valuable lives. From a very large experience in its use, I am satisfied, fully satisfied, that if we depend more on the early use of the lancet in the congestive and inflammatory states of many diseases, our practice would be more successful than it now is. At the present time there is too exclusive reliance upon medicines affecting the nervous and vascular systems, which act with less efficiency and are less prompt. It is, in my opinion, a very important subject, and I feel assured that ere long the lancet will be more freely used than it is now. In the congestive chills preceding inflammatory diseases, and in the cold stages of intermittents, I have frequently broken up the paroxysm, and relieved the patient by the lancet alone."

He advocated strongly the cultivation of professional *esprit de corps.* This had developed to a very remarkable degree since he was a young man, and he gave the credit to the American Medical Association. He concluded his address with the following sentiment:

"At the close of a long life, one devoted unreservedly to the study and practice of medicine, I will say that notwithstanding its uncertainties, its fatigues, its anxieties, its bitter disappointments, I am completely satisfied that in no other career can a man more fully accomplish his whole duty to God and to his fellowmen; so that when life here is ended, it can be truly said of him as—be it said with all reverence—was said of Him whom we should all imitate, *pertransivit benefaciendo*—he went about doing good.

Trusting that our proceedings may be both harmonious and profitable to us all; and thanking you again for the honor you have conferred upon me, I sincerely hope that the recollections we shall carry home with us will be both agreeable and lasting."

At the close of Dr. Atlee's address the meeting adjourned till 1:30 on the 6th. The afternoon was devoted to the usual meetings of the different sections. The attendance was large and the interest in the work was keen and well sustained. We shall commence a synopsis of the sections in our next.

---

McDaniel's Method of Artificial Respiration.—The *New Orleans Medical and Surgical Journal* calls attention to a method of artificial respiration discovered by Dr. A. McDaniel, of Alabama, and, very justly, we think, after having read a description of it, complain that it has not received merited recognition by the profession. That the fault has not lain with the discoverer is evident from the fact that he read a paper describing it before the American Medical Association, in 1869, and another before the Alabama State Medical Society, in 1879. The method is in many respects an improvement on Marshall Hall's and Sylvester's methods; it is especially adapted to small patients. We give a description of it in the author's own words, as copied from our contemporary:

"After the invention of the spirometer, by Hutchinson, it was soon ascertained that the capacity of the chest is greater in the erect form than in any reclined or recumbent position. This is a great fact for physiology, for pathology, and for therapeutics. The chest is a cylinder, and the diaphragm is a piston whose pump motion varies the chest capacity and causes an ingress and egress of air. In the recumbent position the liver and other contents of the abdomen press upon the diaphragm and diminish the chest capacity. In changing from the recumbent to the erect position, this pressure is gradually removed and the chest capacity is increased. It is obvious that all that is necessary to cause air to enter the lungs is to change the patient from any recumbent or any reclined position to the erect one; and all that is necessary to cause the air to pass out of the lungs is to move the patient back from the erect to any inclined or recumbent position. But I have discovered that the increase of capacity in the chest is slow and small in moving from the recumbent position to an elevation of forty-five degrees, and rapid in ascending from forty-five degrees to the erect position. It is, therefore, not essential in practicing artificial respiration to move the patient through the whole range from recumbency to erectness, but is sufficient to use only the upper half of this range, merely moving the patient from a forward inclination of forty-five degrees to the erect position and back again. Every upward and backward movement produces an inspiration, and every forward and downward movement an expiration, and the two together a complete respiratory act. By regularly repeating these acts, artificial respiration is rythmically performed, and can be prolonged at will. Anyone will find that if he leans forward from the erect position to an inclination of say forty-five degrees, he will mechanically and involuntarily expire, and if he moves back to the erect position he will mechanically and involuntarily perform inspiration. He cannot, by any power or volition, prevent the result or reverse it. This simple movement upward and backward to the erect position, and downward and forward to a sufficiently inclined position, regularly repeated, constitutes my proposed new method of artificial respiration."

---

A Word on the Treatment of Syphilis.—We are gradually growing to look with less and less dread and caution upon the *disastrous* effects of mercury when syphilis does *not* exist, and as we do so, we are less afraid to employ it.

It would seem, therefore, that whenever a patient comes under observation who confesses to having had, or where there exists any reasons to suspect that he or she may have had syphilis at some period more or less remote, we are justified in giving specific treatment.

We have seen an eight-ounce mixture, containing $\frac{1}{8}$ grain of the biniodide of mercury and 80 grains of iodide of potassium taken by a patient in whom there was only the *slightest* possible suspicion of a remote infection, without any result, *good* or *bad*, being manifest.

While we do not recommend the *indiscriminate* use of mercury and potassium, yet we hold that the ramifications of syphilis are so numerous and so intricate that in many obscure and obstinate cases of departure from health, where we can *carefully* watch their effects, they will often prove useful where all other means fail.

These words are suggested by a case recently reported by Dr. R. B. Davy in the *Cin. Lan. and Clin.*, March 21, 1883.

The patient was a highly respectable gentleman, aged 70, who had an ugly and infractable ulcer on his leg, which resisted all treatment until he was given iodide of potassium and bichloride of mercury, when it kindly and rapidly healed.

He then admitted having had a chancre fifty years before, followed by constitutional symptoms, and that every ten years since he had return of the manifestations, which always yielded readily to specific treatment.

By bearing in mind the great prevalence of the syphilitic poison, its liability to recur, the profound influence it exerts on diseased conditions, and the comparative innocence of *carefully* watched specific treatment, we will frequently be enabled to secure good results where hitherto we have failed.—*Editorial in Medical and Surgical Reporter, April* 21.

---

Vomiting of Pregnancy.—The following drugs have been recommended for this distressing symptom, which we here arrange alphabetically rather than in the order of their relative importance:

Arsenic, in the form of Fowler's solution, in drop doses, given before meals, is often of great advantage.

Atropia has been highly recommended for the vomiting of pregnancy, in the dose of 1-120 of a grain, injected subcutaneously in the epigastric region. It is said to arrest it promptly and permanently after other remedies have failed.

Bismuth, subnitrate, in ten-grain doses, combined with ¼ grain carbolic acid, mixed with a suitable adjuvant, to be taken three or four times daily.

Calumbo, in tincture, dose 5 to 10 drops; in infusion, teaspoonful.

Cerium, oxalate, dose 2 to 5 grains. Usually the best effects are produced after several day's use.—Sir James Simpson.

Champagne, tablespoonful doses with ice, every fifteen minutes.

Chloral hydrate, with bromide of potassium, 10 grains of each at night when the symptom first develops.—W. C. Burke.

Copper, sulphate, 1-20 grain three times daily.

Hydrocyanic acid, dilute, three-drop doses once in four hours.

Iodine, tincture, drop doses every hour or two.

Nux vomica, tincture, drop doses every hour or two.

Pepsin, five to ten-grain doses.—*Med. Bulletin, April.*

---

Sterility and Stenosis.—In a recent clinical lecture, Dr. Goodell, speaking of a case of anteflection with stenosis causing dysmenorrhœa and sterility, says:

"From my observation I should say that marriage is poison to a woman unless she has children. Nature intended that the womb should by pregnancy have a respite from the monthly congestion of the menses. At the end of nine months the woman gives birth to a child and suckles it, and this also gives rest to the womb. If now there is a stenosis of the cervical canal, she cannot become pregnant; the congestion grows worse at each period, and finally becomes continuous and pathological. Marriage adds to this chronic condition the congestions resulting from the marital relations, as coition, etc., and things go on from bad to worse, until the woman may at last become a confirmed invalid."

His treatment of the stenosis was by rapid dilatation, which he strongly recommends as against a cutting operation. With the exception of two cases in which the os was torn (not, however, with any ill results,) he has never had any accident in one hundred and thirty recorded cases of dilatation, while he has lost a patient from the cutting operation, and believes that many women have died from that treatment.—*Gaillard's Med. Jour.*

---

RAPID DILATATION OF THE CERVIX UTERI.—In a paper read before a recent meeting of the Medical Society of the County of Kings, Dr. John Ball called attention to a report submitted by him in 1873, in which was contained some quite remarkable results in stricture, chronic endo-cervicitis, conical cervix, flexions, sterility, etc., by rapid dilatation of the cervix. He now reports that in the interim of ten years his experience has confirmed him in the views then expressed. In the treatment of such cases, rapid dilatation has entirely superceded tents, the knife, scissors, curette, etc., in his practice. He reports additional cases of dysmenorrhœa, chronic cervical endometritis, menorrhagia and metrorrhagia, in which this method of relief was resorted to with very remarkable success. The operation is performed under an anæsthetic. The dilator having been carefully introduced, is operated by means of a screw, and the canal divulged in all directions to the extent of the capacity of the instrument. The pessary is then introduced and kept in position for a week, the patient being kept on her back the while. Dr. Ball considers his plan quite as safe as any other successful method.—*Med. Age, April* 25.

---

GYNÆCOLOGICAL POINTS.—The following points are culled from the lectures of Prof. W. Goodell, in the University Hospital: Dr. Goodell does not operate in laceration of the cervix, if the sides of tear are in apposition—that is, lie parallel and are not turned up. In erosion of the cervix he recommends the local application of collodion in which iodine has been dissolved, or the strong tincture of iodine may be used. In carcinoma of the uterus Dr. Goodell applies locally the tampon soaked in a glycerole, and gives constitutionally ten drops of Fowler's solution before meals for the cancerous cachexia and twenty drops fl. ext. ergot several times a day to prevent too much bleeding. After every operation on the uterus Dr. Goodell applies a tampon, cup-shaped, in which glycerine is poured. He also instructs the patient how to do this. Dr. Goodell's favorite local applications for endometritis and other similar affections of the uterus are: 1. A mixture of one ounce each of iodine, chloral, and carbolic acid. 2. One drachm of pure carbolic acid to one ounce of glycerine. 3. A saturated compound tincture of iodine. A solution of nitrate of silver of one drachm to the ounce. —*Can. Lancet, Mar.*

---

CASE OF OVARIOTOMY IN WHICH THE EXPANDED BLADDER WAS WOUNDED.—*Gaillard's Med. Jour.*—Dr. Walter F. Atlee reports, in the *American Journal of the Medical Sciences*, a case in which ovariotomy was performed in a woman, aged fifty-six years, in whom the bladder was accidentally opened. In order to close the tear in the bladder, a large gum catheter was passed through the urethra and then into the orifice, and after an assistant had taken a firm hold of the bladder between the thumb and forefinger on each side, it was withdrawn. The edges of the tear in the the bladder were then pushed in and invaginated. A thick plaited silk thread was chosen, having a needle at each end; one needle was passed directly from before backward through the walls of the bladder on one side, about one-quarter inch from the tear, and the other in a like manner on the other side, and then the thread was tied very firmly and cut off close to the knot. After cleaning carefully the abdominal cavity of urine, blood and cystic contents, the wound in its walls was sewed up and the usual dressing applied. Recovery took place.

---

MENINGITIS IN CHILDREN.—*Can. Lancet.*—Dr. Vovard (*Jour. de Medecine, Bordeaux,* Nov., 1882,) claims good results both in tubercular and non tubercular meningitis of children from potassium iodide internally and the application of olium tiglii to the scalp. The head is shaved, croton oil applied, and after the pustules have appeared they are smeared with an irritating cerate. Hebra and others have had similar results from the application of antimony ointment.

Asafœtida in the Treatmtnt of Abortion.—In the *Journal de Medicine de Paris* of December 16, 1882, are collected the results obtained by several observers in the prevention of abortion and premature labor by asafœtida. Dr. Laferla, acting upon the theory that the death of the fœtus was owing to an asthenic condition of the uterus, administered the drug in a number of instances. In nearly ninety per cent. of the cases so treated, the patients (who had aborted from two to five times in former pregnancies) went on to full term. Drs. Giordano and Carzani announce equally favorable results, though the number of their cases was smaller. The latter prescribes the drug in pill form in doses of one and a half grain twice a day, gradually increased to twelve grains per diem. Dr. Gourgnes recommends the administration of asafœtida, in emulsion with the yelk of an egg, by the rectum.—*Med. Record, March* 24.

---

Cauterization of the Clitoris in Hysteria.—*Virhow's Archiv.—Med. Times.*—The late Professor Friedreich, shortly before his death had prepared a paper, which has since been published, on this subject. In many cases of obstinate hysterical affections he has found that cauterization of the clitoris by nitrate of silver has had the most beneficial effects. The cauterization must be severe, as slight superficial cauterization tends rather to aggravate the disease. The pain is at first severe, and during it the patient must remain in bed. Among the cases which he gives as cured with extreme rapidity by this method are —one of paraplegia, which had lasted for a year and a half; hysterical aphonia, lasting for two years; glossoplegia, lasting for four months; tonic spasm of the spinal accessory, lasting for seven months; and several cases of general severe hysterical convulsions.

---

Menopause.—*Med. Review.*—Flatulence and fluttering or palpitation at the pit of the stomach, which are so often distressing to women at the menopause, are relieved, Dr. A. A. Smith asserts (*N. Y. Med. Jour.*), by doses of one-fiftieth of a grain of the extract of calabar bean repeated every half hour for six or eight doses.

Treatment of Placenta Previa.—Supported by an experience of forty-six cases of placenta previa, Hofmeier advocates early interference. As soon as pains begin, resort to version, according to the method of Braxton Hicks, and not lose time with the tampon, which latter does not always stop the hemorrhage and further increases the danger of infection. In case of placenta previa centralis, where the cervix still remains and the os externum is narrow. the author advises, in the event of profuse hemorrhage, the perforation of the placenta and an attempt at delivery of one foot. During extraction, which must necessarily be very slow, ergot is administered. Post partum, the uterus must be washed out with a five per cent. solution acid, carbol. His results are good. In forty-six cases he counts only five deaths.—*Zeitschr. f. Geo. Gyn.* —*Therap. Gaz.*

---

Sassafras in Rhus Poisoning.—Dr. R. L Hinton claims that sassafras tea is almost a specific for the rash produced by poison oak. This is an infusion of the bark of the red sassafras. The diseased parts are covered with compresses soaked in the cold infusion, while internally there is administered the infusion warmed, sugared, and with milk, according to the taste.—*Medical Record, April* 14.

